# Long-term TE persistence even without beneficial insertion

**DOI:** 10.1186/s12864-021-07568-4

**Published:** 2021-04-12

**Authors:** Stefan C. Kremer, Stefan Linquist, Brent Saylor, Tyler A. Elliott, T. Ryan Gregory, Karl Cottenie

**Affiliations:** 1 School of Computer Science, University of Guelph, Guelph, ON N1G 2W1 Canada; 2 Department of Philosophy, University of Guelph, Guelph, ON N1G 2W1 Canada; 3 Department of Integrative Biology, University of Guelph, Guelph, ON N1G 2W1 Canada; 4 Centre for Biodiversity Genomics, University of Guelph, Guelph, ON N1G 2W1 Canada

## Abstract

This correspondence responds to the critique by Butler et al. (BMC Genomics 22:241, 2021) of our recent paper on transposable element (TE) persistence. We address the three main objections raised by Butler et al. After running a series of additional simulations that were inspired by the authors’ criticisms, we are able to present a more nuanced understanding of the conditions that generate long-term persistence.

## Background

The critique by Butler et al. [1] of our recent paper on transposable element (TE) persistence underscores the importance of replication in science. Our somewhat surprising result, that TEs can persist for many generations in an asexual population despite having no net beneficial effect on host fitness, deserves scrutiny because it suggests a novel mechanism by which TEs might persist in nature, that we described as form of “ecosystem engineering” within the genome [2]. Butler et al. raise three objections to our study. First, they note that we were insufficiently precise about the effects of TE insertion on host fitness in describing the aims and results of our model. Second, they argue that the rates of beneficial insertion that we chose for our simulations were artificially high. Third, they admirably conducted a series of experiments using our software and found that long-term persistence is sometimes conditional on the rate of beneficial TE insertion. We are grateful to the authors for these criticisms, they have prompted us to further explore our model and its dynamics. One important discovery was a slight error in the conditions under which we reported long-term persistence in our previous paper [2], which fortunately does not affect our general result. More important perhaps, after running a series of additional simulations that were inspired by the authors’ criticisms, we have a more nuanced understanding of the conditions that generate long-term persistence. Essentially, both we and our critics are partially correct. As we explain below, Butler et al. are correct that a high rate of beneficial insertion can promote long-term TE survival in at least some cases. However, we also discovered long-term persistence when the rate of beneficial insertion is low, even zero. In yet other conditions, beneficial insertion rate shows no consistent relationship to long-term persistence. Hence, we maintain that our TE engineering hypothesis has not been falsified and is one of several mechanisms by which TEs might accumulate and persist over the long term. Before presenting these results, we briefly respond to the first two criticisms of Butler et al.

## Getting clear about “serious deleterious effects” on host fitness

Butler et al. correctly note an ambiguity in our previous description of how TE insertion affects host fitness in our model. Part of our aim was to test the hypothesis that TE persistence requires an average net beneficial effect on host fitness. We sometimes referred to this as the hypothesis that “TE insertions are beneficial and accumulate through positive selection on hosts.” We further described our model as one in which TE insertions had a “serious deleterious effect” on the host. We concede that these statements are each vague and could be misleading.

However, it is difficult to find any general English phrase that captures precisely the effects of TE insertion on host fitness in our model, since this is a function of several parameters that can be described at different levels. Our model consists of a population of organisms, each with a spatially explicit chromosome comprised of either 500 or 5000 genes and a certain amount of non-genic DNA that increases with TE replication. Individual TEs excise and insert with fixed probabilities, sometimes landing in other TEs, but affecting host fitness only when they land within one of the genes.

At the lowest level of description, where an individual TE inserts into a particular gene, effects on host fitness are easy to calculate. This is a function of two parameters. A fixed parameter called *Insertion_effect* specifies the probability that the insertion will be lethal (30%), deleterious (20%), neutral (30%), or beneficial (20%). A variable parameter called *Mutation_effect* specifies the magnitude of either deleterious or positive insertions on host fitness. Each host organism has a baseline fitness value, inherited from its parent, which influences its survival probability when the population undergoes selection. This fitness value is decreased/increased by either 10% (high mutation effect) or 1% (low mutation effect) in cases where *Insertion_effect* is deleterious/beneficial. These effects occur only once, in the generation where the insertion takes place. For example, suppose that a TE lands in a gene, has positive *Insertion_effect*, and a high mutation effect. The host now has a greater chance of surviving selection compared to the average member of its population that varies uniformly between 0 and 10%. Its offspring inherit this slightly elevated fitness-level; however, the beneficial insertion does not continue to increase the relative fitness of its descendants.

We have spoken so far about the effects of a single TE insertion into just one host gene. Each host has 500 or 5000 genes. Hence, a host’s total fitness is a function of its baseline value (inherited from its parent) plus or minus the cumulative effects of *all* TE insertions into its genes. Although 20% of the TE insertions into genes will have a positive effect on host fitness, they are balanced by another 20% that are deleterious. In addition, 30% of the insertions into genes are lethal. This is what we meant by our earlier statement that TE insertion has a “seriously deleterious” effect on host populations. The cumulative effect of TE insertions on an individual host changes with the number of active TEs in its genome. As this number goes from 1 transposon at the beginning of an experiment, to as much as 3000 TEs at the end of some experiments (in which TEs persist), the law of large numbers takes effect. This means that, at later stages of the simulation, as TEs increase in abundance, the likelihood that an organism will experience a net increase in fitness from TE insertion becomes increasingly small. As TEs accumulate in a genome to a large number, TE insertion has no net positive effect on host fitness, yet there is a high (30%) chance that any one of the insertions will be lethal.

With such a strong deleterious effect on the population, it is no surprise that in most of our simulations TEs were either purged or the hosts went extinct. However, in linages where TEs persist over the long term, the number of active TEs can reach several thousand. How is this possible? Our reasoning was that since TEs cannot co-evolve with the host to become less lethal, and since there is no horizontal transmission, nor a net positive effect on fitness, there must be some other process that decreases their chances of landing within a gene. We infer that, in cases of long-term persistence, TEs must be creating neutral insertion sites at a rate that balances the growing number of TEs in the genome. We interpret this as a form of “TE engineering” on the understanding that (as with the broader phenomenon of ecosystem engineering in ecology) this imparts no intention to individual TEs.

## Is a 20% positive insertion rate too high?

Butler et al. suggest that our positive *Insertion_effect* of 20% is unrealistically high. Note that this applies only to TE insertions into genes, as TE insertions elsewhere in the genome have no effect on host fitness. Also, this value should not be confused with the *magnitude* of a single insertion on host fitness, which is specified by the *Mutation_effect* variable. Furthermore, it is important not to mistake the rate of positive insertion with the effects of this variable on host survival.

Our value of 20% positive *Insertion_effect* was based on Eyre-Walker and Keightley [3], who summarize the distribution of fitness effects from multiple studies, reporting 0–15% positive mutation rate in the bacteriophage φX174, and also a > 15% positive mutation rate in *D. melanogaster.* By rounding up to 20%, it is true that our rate exceeded these empirical estimates. However, Butler et al. draw a misleading contrast when they compare our 20% figure to the findings of Rishishwar et al. [4], who estimated that 1.13% of a dataset of ~ 14,000 polymorphic TEs in 1500 human genomes were under positive selection. As we explained in the previous section, our 20% positive *Insertion_effect* variable refers to the *chance* of a single insertion event into a particular gene. It is not equivalent to the cumulative *effects* on an individual host, nor to the proportion of beneficial insertions that will be increasing in the population due to positive selection. To make this point as clear as possible it is necessary to explain *Insertion_effect* in mathematical terms.

Let the proportion of increasing TE incidence in the population be (PITEI). We used a 20% frequency of beneficial insertions in our model, which we can refer to as beneficial probability (BP). The authors of the response paper compare PITEI and BP, and conclude that our frequency of beneficial mutations is too high. Comparing these two percentages is not appropriate because as explained in the previous section, the PITEI is based not only on the degree to which beneficial insertions occur (in the first place), i.e., BP, but also the degree to which they advantage their hosts (which we called *Mutation_effect*) and the probability of this effect increasing their numbers over time compared to the null model.

As we noted earlier, we set our *Mutation_effect* quite low, and that, as a result, the probability of this effect increasing the numbers of a beneficial TE insertion is also quite low.

Specifically:

$$ P\left({s}_i\right)=\frac{L\left({s}_i\right)+{M}_e\times U\left(0,1\right)}{\sum_iL\left({s}_i\right)}\times C $$

Where:

- *P*(*s*_*i*_) is the probability that individual *s*_*i*_ will survive to the next generation,
- *L*(*s*_*i*_) is the likelihood that individual *s*_*i*_ will survive to the next generation,
- *M*_*e*_is the *Mutation_effect* parameter, and
- *C* is the carrying capacity of the environment.

While the survival likelihoods of the population will vary over time depending on the effects of host mutations and TE insertion events, the survival likelihoods are initialized to 1 and generally trend slowly upwards from these. Prior to applying the survival probability, we double the population size by replicating each host individual applying mutations and TE effects, and allowing them to compete with the hosts that survived the previous generation. Thus:

$$ L\left({s}_i\right)\approx 1 $$

$$ \sum \limits_iL\left({s}_i\right)\approx 2C $$

So, the survival probability of the recipient of a beneficial insertion surviving to the next generation, and thereby replicating, resulting in an increase in the number of hosts exhibiting this beneficial insertion is:

$$ P\left({s}_i\right)\approx \frac{1+{M}_e}{2} $$

By contrast an individual without the beneficial TE insertion (our null model) would have:

$$ P\left({s}_i\right)\approx \frac{1}{2} $$

We used 2 values for the *Mutation_effect*: 0.1 and 0.01. Thus, the probability that an individual with a novel beneficial insertion survives even one generation is either 55% (high *Mutation_effect*), or 50.5% (low *Mutation_effect*). After the first generation (all things being equal) each offspring will have an independent 55% or 50.5% chance of surviving. From generation to generation, the number of individuals exhibiting the beneficial mutation will be governed by a series of cascaded binomial distributions.

Of course, there are many other factors which will continue to affect the chance of the beneficial insertion surviving in the population. For example, a single deleterious insertion (which occurs with equal probability to the beneficial insertion) would negate the improvement in the survival likelihood of a host organism, while a single lethal mutation (which occurs with an additional 30% chance) would halt the propagation of the beneficial insertion entirely. Therefore, suggesting that the 20% chance of beneficial mutations would result in a similarly high observation of increased frequency of this mutation in a host population misinterprets and oversimplifies the dynamics of the model.

Butler et al. [1] present a second reason for thinking that our “high” rate of positive insertion might be responsible for long term TE persistence. They point to previous theoretical findings (Le Rouzic et al. [5]) demonstrating that a positive insertion effect as low as 0.05% is sufficient to permit TE “domestication” through positive selection. One important difference, however, is that our model examines an asexual population in which there is no recombination. Under these conditions is it more difficult for selection to increase the frequency of a beneficial insertion, because it cannot be dissociated from deleterious ones. Setting this issue aside, in retrospect we should have explored the effects of variable positive insertion rates on long-term TE persistence, as Butler et al. [1] did in their experiments, and as we shall do presently.

## Is long-term persistence contingent on positive insertion effect?

Butler et al. [1] replicated a subset of the experiments reported in our previous paper, selecting those in which we claimed to have observed long-term TE persistence. They provide evidence that under those parameter settings, long-term persistence is conditional on a positive insertion effect. We were initially puzzled by some discrepancies between the two sets of results. This prompted us to replicate our own previous experiments, and eventually to revisit our earlier results. This led us to discover a mistake in our earlier paper that might have gone undetected were it not for Butler et al.’s replication.

In our previous paper, we thought that we had independently varied 8 parameters, creating 256 unique configurations with 3 simulations each. In fact, we did vary 8 parameters, but *Insertion_bias* was varied dependently on *TE_progeny*, *TE_Excision_rate*, *TE_death_rate* and *Carrying_capacity*. So, we in fact ran only 128 unique configurations, with 6 simulations each. This means that some of the possible combinations explored by Butler et al. were not in fact replications of our original study, but instead novel experiments. It also means that some of the conditions under which we found long-term persistence were not in fact replicated by Butler et al. We apologize to Butler et al. and are relieved our mistake did not impact the value of their contributions to this debate.

Inspired by the work of the correspondence paper we have conducted a new set of experiments varying the value of the *Insertion_effect* and found the result quite surprising. Specifically, we selected 11 conditions in which we had sometimes observed TE persistence: Table 1.

**Table 1:** Experimental conditions in which we previously observed TE persistence

TE_progeny	TE_excision_rate	TE_death_rate	Insertion_bias	Corrected_mutation_rate	NP_BP	Mutation_effect	Carrying_capacity
H	L	L	L	H	L	L	L
L	L	H	H	L	H	H	H
L	L	H	L	L	H	H	H
L	L	L	H	L	H	H	H
L	L	H	H	L	H	H	L
L	L	L	H	L	H	H	L
L	L	L	H	L	H	L	L
L	L	H	H	L	L	H	H
L	L	L	H	L	L	H	H
L	L	L	H	L	L	H	L
L	L	L	L	L	L	H	L

Then, we followed the lead of the correspondence paper, and ran simulations with the same 6 different probabilities of beneficial mutations: 20% (as in our original simulations), 15, 10, 5, 1, and 0%. Just as in the correspondence paper and our original work, the probability of lethal mutations and mildly deleterious mutations were fixed at 30 and 20% respectively, while the rate of neutral mutations was set to make the total 100%. Due to the time-consuming nature of the simulations, we only ran 3 instances of each parameter set and beneficial mutation rate combination, for a total of 11x6x3 = 198 experiments.

**Fig. 1:**
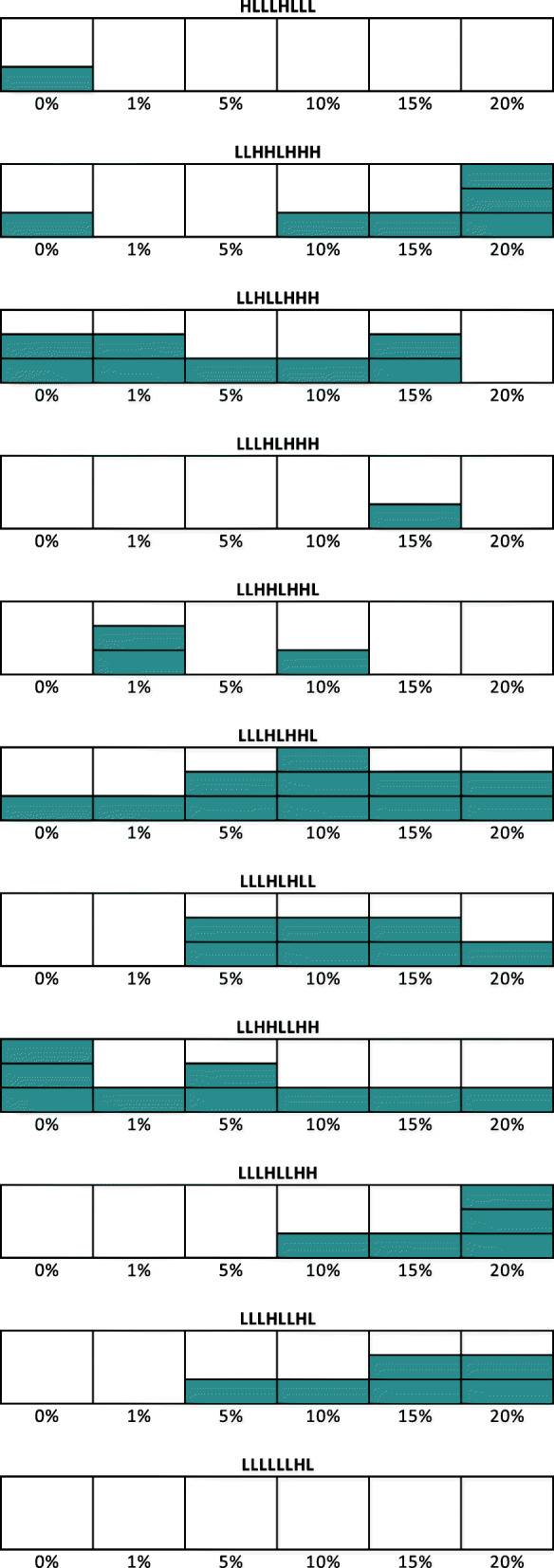
Persistence under 11 experimental conditions. The experimental conditions are indicated at the top of each illustration and correspond to Table 1. The horizontal axis indicates the probability of beneficial insertions. Three repetitions of each condition were performed, and the height of the green bars indicates the number of repetitions which resulted in persistence

We expected to see a diminishing number of instances of TE persistence as the beneficial mutation rate decreased, and no TE persistence in the case of 0% beneficial mutations. The results, however, surprised us (Fig. 1).

We make the following observations:

1. As in the simulations reported in our original paper, the majority of outcomes do not show a persistence of TEs. Instead, the reproduction of TEs is often too low to be sustainable and the TEs become extinct, or alternately, the TEs proliferate at such a rate that they frequently insert into genes with deleterious or fatal effects leading to the extinction of the hosts.
2. There were 57 of 198 simulations in which TEs persisted. To our surprise, TE persistence occurred at *all* levels of beneficial mutation rate, including zero (which is actually also observed in the correspondence paper’s Fig. 1 for the LLLH-LHHL case). Specifically, TE persistence occurred in 8 of 33 simulations with 0% beneficial mutations, 6 of 33 simulations with 1% beneficial mutations, 8 of 33 simulations with 5% beneficial mutations, 11 of 33 simulations with 10% beneficial mutations, 12 of 33 simulations with 15% beneficial mutations, and 12 of 33 simulations with 20% beneficial mutations.
3. In the simulations reported in our original paper we found that only certain parameter combinations were conducive to TE persistence. In these new simulations we found that there may be an ideal beneficial mutation rate for each parameter setting, where certain settings favour a low beneficial mutation rate and others favour a high beneficial mutation rate. In particular, the HLLLHLLL, LLHLLHHH, LLHHLHHL, LLHHLLHH, configurations yield TE persistence with lower valued, beneficial mutation, while the LLHHLHHH, LLLHLHHH, LLLHLHLL, LLLHLLHH and LLLHLLHL conditions yield TE persistence with higher-valued beneficial mutation, and LLLHLHHL seems to favour the middle of the range. The final condition yielded not TE persistence in these new trials.

From our results, we conclude that there are simulation conditions that favour TE persistence. To our surprise, higher beneficial mutation rates do not necessarily improve TE persistence, instead there seems to be a “sweet spot” mutation rate that varies from parameterization to parameterization. We speculate that each experimental condition results in a different TE proliferation rate and that that proliferation rate can be modulated by the appropriate beneficial mutation rate to ensure enough TEs to avoid TE extinction, but not so much as to overwhelm the host.

## Conclusion

We take this exchange to be a case study in the value of scientific replication. Our initial results were unexpected and prompted the suggestion of a new mechanism by which TEs might persist over the long-term. In replicating our experiments, Butler et al. [1] not only alerted our attention to an oversight in our original paper, but also have potentially refined our general understanding of the relationship between the frequency of positive TE insertion and the likelihood of TE survival. Our combined results suggest that perhaps there is an optimum level of positive mutation rate that contributes to long term persistence, but that this variable is sensitive to a variety of background conditions. Nonetheless, it is a striking fact that, both in Butler et al.’s experiments and our own, long-term TE persistence can occur when the rate of beneficial insertion is zero. We therefore maintain that the TE-engineering hypothesis remains a viable candidate for explaining how TEs might persist over the long term.

We encourage others to explore the provided software and continue to challenge our work.

## Data Availability

The software described below and used to generate the results described is available at: https://github.com/stefan-c-kremer/TE_World2. All datasets were generated from this software and can be regenerated by interested readers.

## Abbreviations

TE: Transposable element
PITEI: Proportion of increasing TE incidence in the population
BP: Beneficial probability of TE insertions
